# An efficient multilevel security architecture for blockchain-based IoT networks using principles of cellular automata

**DOI:** 10.7717/peerj-cs.989

**Published:** 2022-05-25

**Authors:** Fasila Ali, Sheena Mathew

**Affiliations:** School of Engineering, Cochin University of Science and Technology, Ernakulam, Kerala, India

**Keywords:** IoT security, ChaCha20 cipher, Blockchain for IoT security, Cellular automata based Random number generation, Dynamic group key generation

## Abstract

The tremendous increase in the use of Internet of Things (IoT) has made an impact worldwide by changing the mode of day-to-day life. Like any other application, IoT based networks also have to be protected since the data produced consist of sensitive information. Existing algorithms for providing security in such networks do not consider all the security objectives. Starting from the sensing of data from IoT environment, the data have to be protected from several types of attacks. Also, the authentication of involved entities, integrity of data, access control and confidentiality are to be achieved. This work proposes a novel security architecture for IoT based distributed applications. The architecture uses the best known lightweight cipher ChaCha20. Principles of cellular automata are applied for random number generation to attain more security and randomness. Double encryption ensures multilevel protection of data during the data uploading and storing phases. Providing encryption based on dynamic session keys guarantees the security of the method. It also ensures secure data sharing, mutual authentication between communicating entities, fast execution, user authentication and message integrity. The IoT device connected to a gateway node has to complete registration phase successfully. Subsequently, each time a data transfer between the device and gateway node takes place, mutual authentication phase is executed. Blockchain network used at the edge level ensures authentication of participating nodes and hence, unintended modification of data is prevented. The proposed architecture proves to be efficient in terms of throughput, execution time and resistance to various security attacks.

## Introduction

Internet of Things (IoT) applications connect huge number of devices worldwide and produce tremendous volume of data every day. As per definition in Pethuru & Anupama (2017), IoT can be considered as a dynamic global infrastructure with capabilities like sensing data from surroundings, self-configuring, interoperability, interfacing, taking actions based on data processing, *etc*. These data contain information with varying levels of sensitivity. For example, IoT devices connected to healthcare applications produce sensitive data related to individual persons. Almost all of the IoT based networks deal with sensitive data nowadays which brings up the need for efficient security algorithms. The need for security in IoT systems is depicted in Koduru, Reddy & Padala (2019). Conventional cryptographic algorithms that are proved to be secure cannot be applied in IoT networks since the devices involved are constrained in terms of computational capabilities and resource constraints (Qasaimeh, Al-Qassas & Ababneh, 2021). Usually, data are uploaded to cloud storage by the gateway devices (Mrabet et al., 2020). To protect data from unintended users, some form of encoding has to be done. Conventional encryption algorithms are of two types-symmetric and asymmetric. In symmetric algorithm, the key used for encrypting data is shared with the recipient node to get access. IoT based networks prefer symmetric encryption since asymmetric techniques are based on complex mathematical computations. Another criterion divides the encryption algorithms into block cipher and stream cipher (Mohammad et al., 2021). Block cipher takes the input data in blocks and produces cipher text in blocks, whereas, stream cipher takes plain text as stream of bytes. IoT based networks prefer lightweight stream ciphers (Mohammad et al., 2021). By the term “lightweight”, it is meant that the operations involved are lightweight in nature. Several lightweight stream ciphers have already been proposed. The National Institute of Standards and Technology (NIST) of the United States initiated a project (Sonmez et al., 2021) exclusively for finding best lightweight cryptography methods. In the latest report submitted by the project team, it is suggested to use symmetric ciphers for providing high grade security in constrained devices. A resource constrained communication system has to incorporate any lightweight encryption algorithm to achieve confidentiality.

ECRYPT eSTREAM project (Robshaw, 2008) supported by European Commission, evaluated several lightweight ciphers. The study was conducted on two categories of stream ciphers-first profile was based on software performance and second profile was based on hardware performance. For better performance in software implementations, stream ciphers are preferred. Various statistical tests like randomness test were conducted on candidate stream ciphers and the finalists were selected. The major final level candidate algorithms from eSTREAM project are Grain, HC-128, Salsa 20/12, Sosemanuk *etc*. Based on these studies by NIST and eSTREAM projects, the successful stream cipher candidates were shortlisted. The list of final round candidates is provided as File S3. Salsa20 proved to be secure and unbreakable. Tests like truncated differential cryptanalysis, non-randomness and differential cryptanalysis were done on Salsa20 and it was successfully verified up to 12 rounds (Barbero, Bellini & Makarim, 2021). It achieved the highest weighted voting score among all Profile 1 algorithms. ChaCha20 is a variant of Salsa20 and this was developed for achieving better diffusion and performance compared to Salsa20. ChaCha20 encryption is approximately five times faster than AES on low end devices. So, ChaCha20 is used by Google as a replacement to conventional RC4 algorithm (Barbero, Bellini & Makarim, 2021). Its major advantage is the design based on ARX (Addition, Rotation and XOR) technique and this helps ChaCha20 cipher to provide better performance with less complex structure and resistance to several attacks. Due to these potential benefits of ChaCha20, it is selected as the lightweight encryption technique in the proposed scheme.

After encryption, data will be uploaded to the storage space such as cloud. The users who upload the data knowingly or unknowingly to cloud have to trust the cloud administrator. However, the administrator need not always be trustworthy which again increases the stress and worries of data owners. The data uploaded have to be protected from all kinds of unintended parties and at the same time, intended and authenticated users have to access the data without any obstacles. For such storage systems, achieving security objectives such as authentication, confidentiality and integrity becomes vital. One of the recent paradigms, known as blockchain is advocated for providing IoT security (Khanal et al., 2022). Blockchain can be used to provide authentication of nodes and integrity of data uploaded. Even though, it was proposed for crypto-currency based applications, it is not restricted to that. Distributed applications which handle big data evolved from IoT based environments can be supported by using a distributed ledger like blockchain (Khanal et al., 2022). The default cryptographic credentials present in the blockchain ensure the authentication of participating nodes (Hyperledger Fabric: A Blockchain Platform for the Enterprise, 2020). Since these are based on asymmetric cryptographic primitives, it is not preferred to apply blockchain at the lower level of IoT networks which contain resource constrained devices. Rather, the gateway nodes with more computational resources compared to the physical layer devices can form a blockchain (Khanal et al., 2022). In addition to this, the trustworthiness of a centralized third party will not be a problem if it is replaced by a distributed paradigm like blockchain (Khanal et al., 2022). Also, when blockchain is used, unwanted modification to data is never possible without the consent of participants.

Before beginning any communication, the two parties who exchange data are to be authenticated (Yeh et al., 2011). This mutual authentication phase is not considered by majority of the existing security frameworks. The proposed method has a mutual authentication phase between the IoT device and the gateway device. The session is further protected by encrypting all the data in the current session by using a group key which is provided to the device by the gateway node after its successful registration. Conventional password based login mechanisms are not always convenient for an IoT environment which will upload the data continuously (Gope & Sikdar, 2019). Also, the key used for encryption has to be shared with the data requesting node in a secure channel. All of these security factors are being covered in the proposed method. The main contribution of this work is that it provides a new security framework considering all these security objectives. To enhance the level of security, principles of cellular automata are adopted in the random number generation steps of the encryption and mutual authentication phases.

The rest of this paper is arranged as follows. The following section focus on related works that describe existing algorithms and techniques for providing IoT security. This is followed by the detailed explanation of the proposed method. The implementation details and results are given in the next section. Finally, security analysis of the proposed method is provided.

## Related Works

In this section, state-of-the-art of the authentication algorithms and encryption algorithms in current IoT based networks are discussed.

### Authentication

Authentication is the process by which the entity which is involved in the communication is proved to be the same which it claims. For lightweight IoT environments, several authentication methods were already proposed. Authentication protocols proposed in Yeh et al. (2011) and Shi & Gong (2013) are based on elliptic curve cryptography which is based on asymmetric cryptosystems. This involves more computation which is not suitable for lightweight environments. Also, the method in Yeh et al. (2011) does not consider mutual authentication. The protocols for authentication given in Gope & Sikdar (2019), Turkanovic, Brumen & Holbl (2014) and Farash et al. (2016) are suitable for heterogeneous wireless sensor networks. However, these are prone to password guessing, smart card theft and node impersonation attacks. The device authentication protocol given in Amin et al. (2016) was based on public and private key pairs. However, cryptosystems based on asymmetric techniques are not suitable for constrained devices, as they involve more computational overhead and complexity. The work in Shen et al. (2017) proposed a secure authentication protocol based on tree-based signature in hierarchical attribute authorization structures. Even though it is resistant to a wide range of attacks, it is not suitable to resource constrained environments because of more computations. An authentication scheme based on two factors is proposed in Li, Liu & Nepal (2017), in which mutual authentication between the device and a server is discussed in detail. The steps involve fuzzy extractor generation and this leads to computational overhead. Another important aspect in IoT security is the Group Key Management. An approach for proper group key management was given in Kung & Hsiao (2018) and this is based on hash functions and lightweight cryptographic operations. Operations such as bitwise-XOR and one way hash functions are lightweight and are suitable for constrained devices. Kerberos is a third party authentication protocol which is conventional in use. But, it is not preferred for IoT applications. Several password based authentication schemes are proposed. Such an authentication scheme was proposed in Zhao, Shiming & Liehui (2018) and this focuses on systems with multiple servers. The basic idea used for authentication is again elliptic curve cryptography and this provides security against impersonation attack and password guessing attack. But, as mentioned already, this is an asymmetric cryptographic technique which makes it not preferable for the IoT environments. An authentication mechanism based on Datagram Transport Layer Security (DTLS) is proposed in Shahid et al. (2016). The method is called S3K, Scalable Security with Symmetric Keys. According to this symmetric scheme, for establishing a secure connection between a client and a server, client requests for a key to a trusted third party. The scheme proposed based on DTLS (Shahid et al., 2016) uses Constrained Access Protocol (CoAP), in which the constrained set of devices maintains an Access Control List (ACL). But, the involvement of a third party is not always trustworthy. An authentication scheme based on Kerberos and RADIUS (Rigney et al., 2000) is proposed in Pereira, Eliasson & Delsing (2014) for providing authentication in IoT devices using CoAP. The disadvantage of such methods is that the IoT devices with limited capabilities will have to implement additional preparations and infrastructure to implement protocols like Kerberos and RADIUS.

Application-specific authentication schemes are proposed in several works. Radio Frequency Identifier (RFID) based authentication method using elliptic curve cryptography is proposed in Debiao & Sherali (2015). As authors mention, it is a good method in terms of security, but not suitable for all IoT applications. The lightweight authentication protocol described in Jian et al. (2018) is suitable for Social Internet of Things, especially for Vehicle to Grid (V2G) networks. A three factor chaotic map based authentication scheme using biometrics is given in Sandip et al. (2018). First factor is a smart card, secondly, password and finally, personal biometrics. This method eliminates extensive mathematical computations involving Elliptic Curve Cryptography. This method is suitable for authentication in e-healthcare systems. Authors perform the security checks using the analysis tool called ProVerif (Bruno et al., 2021). OAuth2.0 (Jalaluddin et al., 2018) is a framework to provide authentication in IoT device communication. A token based authentication protocol based on OAuth2.0 framework is proposed in Muhammad et al. (2019).

Authentication schemes based on blockchain are introduced in many works. The architecture consists of set of blocks in which one will be the genesis node or root node. This keeps the history of all operations. The concept was first introduced by Nakamoto (2008). If an end node wants to add a new transaction, it performs the computation and signs it with private key. This is broadcasted to other members in peers. Validator nodes perform validation and minor node adds the new block to the chain. The working is depicted in Mohamed et al. (2019). Several applications of blockchain are there. These include identity based management, access control techniques and authentication in agriculture, transportation systems, healthcare applications etc. (Rui et al., 2018; Mgyar, 2017; Ray et al., 2021; Omar et al., 2021; Liang et al., 2017; Subramanian & Thampy, 2021; Ariel et al., 2016; Aran, 2020; Rakesh et al., 2019; Angelos & Konstantinos, 2021). The security benefits of using blockchain are explained clearly in all these works. The cryptographic credentials assigned to the member node always ensure the authentication. Also, once data block is added to the chain, it cannot be modified and this feature ensures message integrity also. Blockchain based access control systems have proved to be secure as could be inferred from the studies done so far. Henceforth, the major research problem is to identify an efficient authentication scheme to ensure mutual authentication between uploading device and the gateway node.

### Lightweight Ciphers

IoT networks are always prone to various kinds of attacks. To provide protection to data, encryption algorithms could be applied. The main objective is to identify fast, efficient and lightweight encryption scheme for uploading data to the cloud securely without losing data owner’s privacy. Mostly, IoT devices are deployed in environments where there is no provision to provide continuous power supply (Qasaimeh, Al-Qassas & Ababneh, 2021). Advanced Encryption Standard (AES) is a conventional secure symmetric cipher. Though AES cannot be applied as such in IoT environments, its lightweight encryption variants (Weize & Selçuk, 2017; Luo et al., 2015; Huang et al., 2006; Benhadjyoussef et al., 2016; Benhadjyoussef et al., 2012) have been proposed for IoT security. However, these works did not consider the authentication of communicating entities. Another scheme for IoT security uses attribute-based encryption and this is capable of ensuring secure access control and data confidentiality simultaneously (Wang et al., 2012; Tokunaga & Blaauw, 2010).

After taking the key and Initialization Vector (IV) as inputs, the stream cipher generates a key stream which is XORed with the plaintext to produce the cipher text. The strength of any stream cipher is in the generated keystream (Subhrajyoti & Bubu, 2020). The randomness in the keystream generation is what brings security and makes it unpredictable. Stream ciphers encrypt stream of data from the plain text and are generally faster compared to block ciphers in hardware implementations (Subhrajyoti & Bubu, 2020). To promote the research and development of lightweight stream ciphers, the eSTREAM project (Robshaw, 2008) started. A detailed comparison study of all existing lightweight stream ciphers is given in Subhrajyoti & Bubu (2020) and Paul (2020). Random number generation is the key part of this keystream generation procedure. For this, cellular automata (CA) principles can be used (Satyabrata, Umashankar & Jyotirmoy, 2019).

CA principles were introduced by Von, Burks & Goldstine (1966) and Ulam (1950). Though CA was proposed to study biological processes, these could also be used to model complex systems. CA has a set of 256 rules which can be either reversible or irreversible. Authors in Subhrajyoti & Bubu (2020) proposed a lightweight cellular automata-based cipher. Data Encryption Standard (DES) based on principles of CA was proposed in Rama et al. (2011). They proved that using CA rules makes the operations involved lighter and simpler. Research given in Pratibha, Niranjan & Manoj (2013) proposes the use of two-dimensional CA rules in encryption of plain text. It uses steganography and cryptography to provide security. CA based Deoxy-ribo Nucleic Acid (DNA) cryptography method is given (Shanmuga et al., 2015). Here, rule 51 from the set of 256 CA rules (Berto & Jacopo, 2012), is used for the binary sequence. According to their study, rule 51 has many features which make it suitable for cryptographic applications. Stream ciphers based on CA were also proposed in many works. CA consists of a number of cells and these cells will be updated based on the values of its neighboring cells (Wolfram, 1986). The transformation takes place based on a specific set of rules. The number of neighboring cells involved in an updating phase depends on the radius of the CA. In the basic mathematical shift registers that are used conventionally in a cipher, bits are simply shifted. However, in case of CA, cell values are transformed simultaneously to get the next set of values. This strengthens the possibility of using CA as a pseudorandom number generator. As proposed by Wolfram (1986) applying rule 30 of a CA, pseudorandom numbers can be generated. The statistical randomness testing procedure and results for the same are given in Wolfram (1986). As it was mentioned before, CA consists of a grid like structure. In a one-dimensional CA (Wolfram, 1986), the cell before the leftmost cell and the cell after the rightmost cell are assumed to hold the value, zero. Values of the current cell, C_i_ at time t+1 are decided by the rule number and the values of that cell at time t, values in the left cell and the right cell. This can be represented as given below.

(1) \begin{eqnarray*}{C}_{\mathrm{i}}^{\mathrm{t+1}}=Ri({C}_{i-1}^{\mathrm{t}},{C}_{\mathrm{ i}}^{\mathrm{t}},{C}_{\mathrm{ i+1}}^{\mathrm{t}}),\text{where Ri is the rule number}.\end{eqnarray*}

Pseudorandom number generation based on CA is explained clearly and analyzed in Wolfram (1986).

There are a few more lightweight stream ciphers based on CA. Trivium (Canniere & Preneel, 2008) is another best stream cipher identified by eSTREAM project. NOCAS (Sandip & Roy, 2011) is CA based stream cipher was developed based on a non-linear 3-neighbourhood CA. CAvium (Sandip, Depdeep & Roy, 2012) is a CA applied version of Trivium, in which the conventional shift registers are replaced with CA rule set. CAR30 (Sourav & Roy, 2013) is a 3-neighbourhood CA based Grain-128 cipher. FResCA (Jimmy & Roy, 2016) is a CA based stream cipher which combined three and four neighborhood CAs. The above mentioned stream ciphers except CAvium was inspired from the design of Grain-128. Attacks on stream ciphers take place by exploiting the algebraic degree and linearity principles of existing shift registers. When shift registers are replaced with CAs, this vulnerability can be reduced. Also, CA based stream ciphers are resistant to fault attacks (Jimmy, Sourav & Roy, 2016). Security of lightweight ciphers has to be analyzed by performing tests such as randomness test, autocorrelation, avalanche effect, etc. The lightweight algorithms based on CA are stronger because of the feature of rule vector selection and it has been proven to be secure. So, in proposed method, principles of CA are used.

Attribute based encryption (ABE) is a type of asymmetric cryptography, which is also known as public key cryptography (PKC) (Goyal et al., 2006). A scheme which combines ABE and functional encryption is given in Sahai & Waters (2004). Security algorithms for message confidentiality, integrity and node authentication in IoT environments based on ABE are discussed in Bethencourt, Sahai & Waters (2007) and Sphurti & Borkar (2017). The number of works based on ABE itself highlights the strength of this method’s applicability in IoT networks. The highlights of using ABE schemes in IoT communication are:

• Several security features can be achieved simultaneously.
• End devices with limited computing resources will be benefitted since the computation involved is less.

ABE scheme is also used in the proposed scheme if the data items to be protected are of lower priority.

After encryption, when the data items are uploaded securely to the cloud. However, here the data owner cannot trust the cloud owner. The users who want to access data from cloud will have to request to the cloud administrator and based on the access control system present, the access will be given. Therefore, if this is hacked by an attacker, security is compromised and everything will be available to attackers. Here the main objective is to solve the problem of trustworthiness of the centralized third parties such as cloud.

Summarizing all the existing works, it is clear that a single framework which considers all the factors such as confidentiality, user authentication, message integrity, mutual authentication and key management, is not present. Finally, the identified research problems can be summarized as follows.

1. How can data be uploaded to the cloud securely without losing data owner’s privacy?
2. How can mutual authentication between uploading device and the gateway node be ensured?
3. How can the problem of untrustworthiness of the centralized third party in conventional IoT applications be solved?

Currently, all communication steps between two entities are encrypted by using the same key. However, if the key is compromised, protection of the entire system is lost. Hence, it is preferred to update the key frequently. Similarly, when a user tries to access data from the cloud, authentication of the requesting user has to be ensured. Also, it has to be ensured that request is granted as per the defined access control specifications. For this, data can be classified based on the relevance. Access to data with higher priority will be restricted only to authorized persons. Also, high level of protection is required for such data. In addition to the major research problems mentioned above, following issues are also addressed in this proposed scheme.

• Is there any solution to eliminate the use of a fixed key for all communication between two entities?
• How access control to confidential data in IoT applications is ensured?
• How are different levels of protection provided for data belonging to different priority levels?

## Proposed Method

The proposed system is able to address all these issues and it is also resistant to several attacks to which existing methods are prone to. Highlights of the work are as follows:

1. A novel security framework is proposed to achieve confidentiality, integrity, authentication, and secure data sharing.
2. The best lightweight cipher known to be secure till date is selected to provide confidentiality.
3. The main random number generation phases are based on principles of cellular automata.
4. Blockchain paradigm is applied to ensure entity authentication.
5. Sessions between gateway device and the IoT device are always protected by using dynamically generated group keys.
6. All the operations by constrained devices are lightweight in nature.
7. Data are classified into two categories based on the relevance. Security algorithms are applied based on this classification.

**Figure 1:**
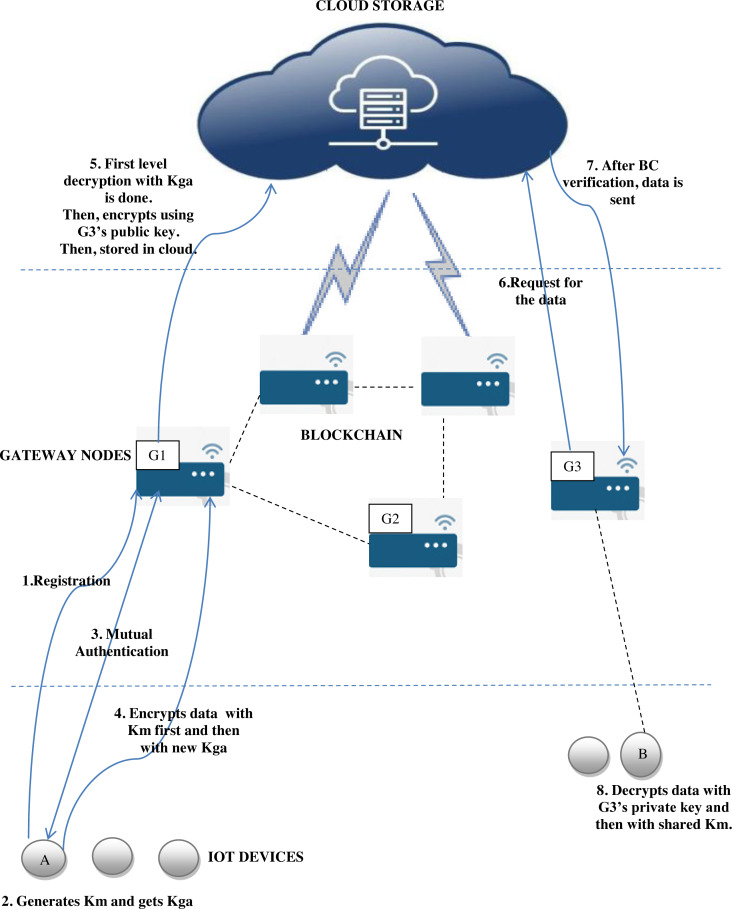
Proposed system workflow.

The proposed architecture consists of four layers. The first layer is an IoT layer with sensing nodes. The second layer consists of gateway devices with more computational capabilities compared to the first level devices. The third layer in existing works which is designated for performing authentication and other control checks is replaced by a blockchain of gateway nodes in the proposed system. The fourth and final layer in the architecture is a cloud to which data are uploaded. The proposed workflow is given in Fig. 1. G1, G2 and G3 are gateway nodes. A is the IoT device. The group key is represented as Kga, Km denotes master key of a particular device and BC denotes blockchain.

Work flow of the proposed system can be explained in following steps:

1. A device when connected to the IoT network has to register with the gateway node.
2. After registration, the device generates the master key Km, to be used for its data encryption. Similarly, gateway node shares the initial group key Kga with device.
3. Device senses data.
4. Device performs mutual authentication with the gateway node.
5. Device encrypts the data using enhanced ChaCha20 stream cipher first with its private master key and then with the group key generated for current session based on cellular automata.
6. Device sends the cipher text to gateway node.
7. Gateway node decrypts it with the group key and classifies the data based on sensitivity to high priority and moderate priority classes. High priority data are to be kept highly confidential. This classification is based on the application and relevance of data.
8. If the data belong to high priority class, these have to be encrypted by the public key of the intended recipient. Otherwise, data items are encrypted by using ABE method.
9. Gateway nodes are members of blockchain. According to the concept of blockchain, any new transaction will have a corresponding hash value and the same will be recorded in the ledger. Similarly, once the new data element is added, the hash value corresponding to this insertion will be stored in the distributed ledger of blockchain.
10. After getting approval from blockchain validating nodes, data are uploaded to the cloud.
11. When data user node requests for the data from cloud, blockchain verifies whether the attributes specified in ABE encryption of data are matching with the requesting node’s attributes.
12. If attributes are matched, data access is permitted and the data item is exchanged in encrypted form.
13. If the data belong to a high priority class, the same will be again in encrypted form (with receiver’s public key). So, the receiver can decrypt the data by using his/her private key.

The workflow can be explained as different phases.

**1. Registration Phase:** Initially, when a new device gets added to an IoT network, it has to register with a particular gateway node to which it is connected. For this, device unique identification details such as MAC address, timing information and location must be provided to the gateway node. Once the device completes the registration process successfully, it can generate a random number which is to be used as the initial key for generating keystream in the lightweight encryption algorithm. Also, the gateway node has a randomly generated, predetermined initial group key which is shared between all devices under that gateway. The same will be passed to the newly added device also.

**2. Data Sensing:** The new device senses data. Before uploading the data to cloud, it has to complete a mutual authentication phase with the gateway node.

**3. Mutual authentication:** This phase consists of a handshaking procedure with four steps.

Step 1. Device sends a message, “Message_1” (encrypted with initial group key) to gateway node, which comprises of its own identifier, Gateway node identifier, current timestamp, randomly generated nonce value.

Step 2. On receiving this, gateway node compares the received timestamp with the timestamp information embedded in the message. If the difference exceeds a threshold, the message is not considered. Gateway node computes the nonce value and compares it with the received nonce. This ensures integrity of message.

Step 3. Gateway node sends a message, “Message_2” (encrypted with initial group key) to the device, which comprises of its own identifier, device identifier, current timestamp, nonce value.

Step 4. On receiving this, device compares the received timestamp with the timestamp information embedded in the message. If the difference exceeds a threshold, the message is discarded. Device computes the nonce value and compares it with the received nonce. This ensures integrity of message.

The equations and representations of two messages, Message_1 and Message_2, exchanged between gateway node and the device in mutual authentication phase are provided in File S5. Once this mutual authentication phase is completed, the device updates the value of group key by using random number generation based on cellular automata rule. Current value of the group key will be assigned to the cells of CA and then, the selected rule will be applied to get the new group key. Here, rule 30 is used, which is a linear one-dimensional cellular automaton rule. Each cell in a CA has two possible states 0 and 1. Rule 30 can be represented as: left XOR (central OR right), where “left” is the left neighbor of current cell and “right” is the right-side neighbor.

**Figure 2:**
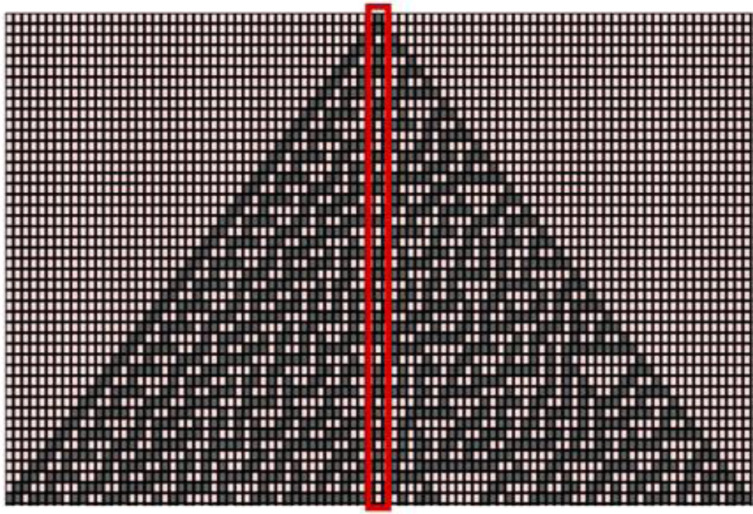
Random number generation using rule 30 of cellular automata.

The aperiodic and chaotic behavior of rule 30 makes it capable to produce complex and random patterns from simple, well-defined rules. To generate random numbers using Rule 30, center column of the generated pattern is taken and a set of n values is randomly selected from the middle column to get a random number of length n, as shown in Fig. 2. Value of n will be the length of key under consideration. For example, key size of ChaCha20 cipher is 256 bits. So, 256 consecutive values will be taken from the pattern produced by CA. The same procedure is used to produce the nonce or initialization vector to be used in ChaCha20 cipher input. In that case, initial contents of the CA will be filled with an initial seed value. Once mutual authentication between device and gateway node is complete and the key values are updated accordingly, device can encrypt and forward the data to gateway node.

**Figure 3:**
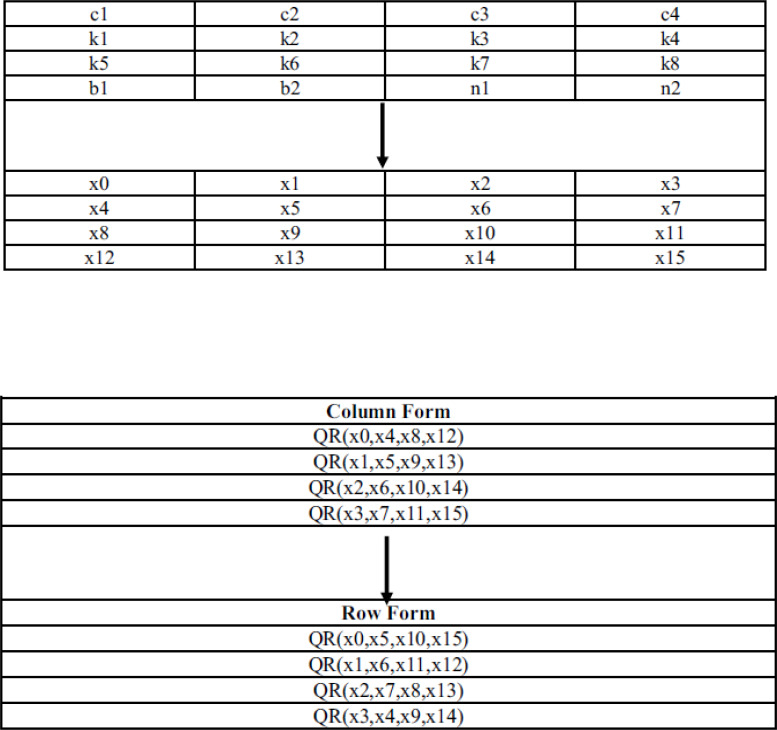
Input and quarter round function of ChaCha 20 cipher.

**4. Encryption and uploading phase:** The device uses the lightweight cipher ChaCha20 (Barbero, Bellini & Makarim, 2021) for encryption. It was proposed by D. J. Bernstein in 2008. It takes an input key of 256 bits, a 64-bit nonce and constants as input and generates a keystream. This keystream is XOR-ed with the plain text input to generate the cipher text. Input size of the ChaCha20 is 512 bits (Mohammed et al., 2021). Input consists of a key sequence of 256 bits length, nonce values and constants. The lightweight procedure consists of an addition (adding of two 32 bits), Exclusive-OR (XOR operation between two 32 bits) and rotation of 32 bits by × bits where × is a constant number (Mohammed et al., 2021). These three lightweight operations form the dual function. Chacha20 variant is proved to be secure so far. ChaCha20 takes 16, 32-bit input words and produces 16, 32-bit output words. The first four words in input are constants. ChaCha20 has 20 rounds with “column” rounds and “diagonal” rounds done alternatively. Each round consists of a “quarter-round” function and this will be run four times producing a different set of words each time. The quarter-round function takes 4, 32-bit words (p, q, r, s) and updates them as follows, where < <  < is a bitwise, left rotation:

p + = q; s ˆ= p; s < <  < = 16;

r + = s; q ˆ= r; q < <  < = 12;

p + = q; s ˆ= p; s < <  < = 8;

r + = s; q ˆ= r; q < <  < = 7;

The 16 words are arranged in a four by four matrix form in which first word occupies the top-left position and the fourth word occupies the top-right position. Then, the quarter-round function is applied four times to the four columns, from left to right in “column” rounds. In “diagonal” rounds, the quarter-round is applied to the top-left diagonal, bottom-right diagonal. This will be followed by the pattern shifted one position to the right, for next three quarter-rounds. To be more specific, in a “column” round, the quarter-round function is applied to the following indices: (0, 4, 8, 12), (1, 5, 9, 13), (2, 6, 10, 14) and (3, 7, 11, 15). In “diagonal” round, it is applied to the indices: (0, 5, 10, 15), (1, 6, 11, 12), (2, 7, 8, 13) and (3, 4, 9, 14). After performing these steps 20 times, the 16 input words are added to the 16 words to produce the 16 output words. Then, these 16 output words are serialized in little-endian order to get the 64 output bytes. Input structure and round function is given in Fig. 3.

Advantages of using ChaCha20 are:

• Simple implementation compared to conventional algorithms like AES.
• Stream cipher based on AXR design.
• Does not need S-boxes. Hence, look up time is very less.
• Proven to be secure and unbreakable to cryptanalytic attacks.

After this first level encryption, it is again encrypted by the new group key value using the same cipher ChaCha20. On receiving this, gateway node decrypts the data with the group key. However, first level protection is not decrypted. Hence, even if the gateway node is tampered at any point of time, the data are not revealed to the attacker. Then, the gateway node categorizes the data based on the application it is maintaining. If the data item is too confidential, there will be an intended recipient. In that case, the current data item in encrypted form is again encrypted by applying public key cryptosystem with recipient’s public key. Hence, this can be decrypted only by the authorized recipient. If the data item is of moderate confidentiality, ABE scheme is applied for encryption. The uploading by the gateway node is marked in the distributed ledger maintained by the blockchain network. This ensures validity and integrity of data always. Only hash of the message is added to the ledger and hence, storage limitation in blockchain is overcome. It also protects the data from unwanted modifications. The built-in cryptographic mechanisms in blockchain ensure the authentication of involved gateway nodes. The hyperledger fabric platform is used in this work and it uses cryptogen tool (Hyperledger Fabric: A Blockchain Platform for the Enterprise, 2020) to create public and private key pairs for newly added gateway nodes. Finally, the data reaches cloud storage securely.

**5. Data access by a node** When another node wants to access the data, it sends request to the gateway node. Access control to a particular data is verified by the blockchain. Access control methods are implemented by using smart contracts. The attributes of the requesting node are compared with the attributes specified in encryption of data in cloud. If both are matching, access is granted. If the data belong to higher priority class, the same will be encrypted with receiver’s public key. Smart contracts implemented in blockchain compares the public key of the requesting gateway node with the public key used for encrypting data in cloud. If both are matching, access is given and the data are moved to the requesting node. If the data belong to moderate class, only ABE will be implemented. On receiving this, both levels of protection are removed by performing decryption by the authenticated recipient node. For performing the decryption of inner level encryption which was done by using the device master key, the key value has to be shared. For this, the proposed method uses a no-share key exchange algorithm (Mahalingam & Fasila, 2020). Requested data item is finally delivered to the recipient node successfully.

## Implementation Details

A test case was developed based on a simple healthcare application which senses patient health information. A registered IoT device and its associated gateway node have to complete mutual authentication phase as explained in the previous section. Algorithms for key exchange and encryption were implemented in Python 2.7. The encrypted data will be uploaded to the cloud storage. Here, the storage used is MongoDB. Blockchain platform used is hyperledger fabric.

As a test case, two organizations were created and an organization has two peers each. A channel is created to share the ledger and organizations are added to this channel. Chaincodes (smart contracts for the blockchain) are written in the programming language, Golang and these are installed to channels. Then, the chaincode has to be instantiated by any member in the network. Required certificates and cryptographic materials are generated using “cryptogen” tool. When the user gateway node is enrolled to blockchain, a private and public key pair is created. Private key will provide authentication in all the future communication from this gateway. Initially, for data sharing, an access control list was maintained. But, this had an overhead of keeping the list of users who will get access. Later, this was replaced by ABE method. Compared to the former, attribute based scheme provided an efficient access control mechanism by simply representing the group of persons who can be given access with a predicate statement. When the data item is requested by any user, blockchain will check whether the user attributes matches with the specified set of attributes. The code for this access control test is written in the smart contract in Go language.

## Results and Discussion

**Figure 4:**
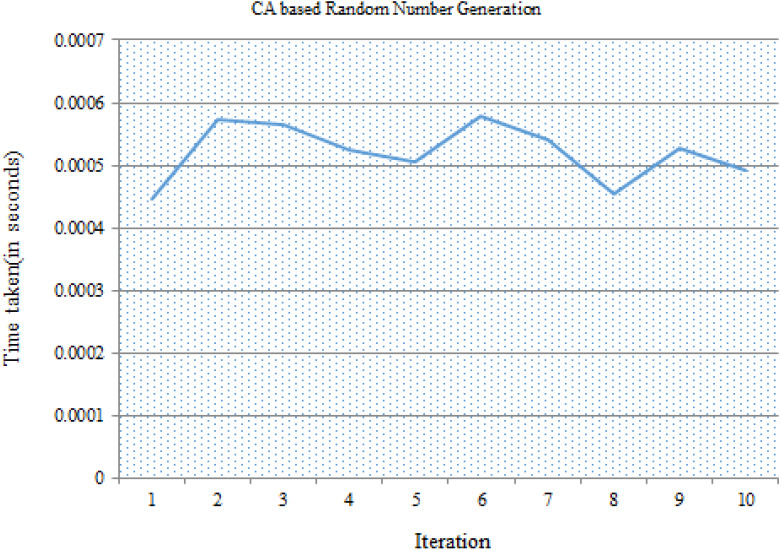
Time taken for random number generation using CA.

The security framework was developed and the performances of different phases were analyzed in detail. IoT device connected to the gateway node has to compute the new group key for encryption for every session. For this, the principles of CA were used. The nonce value with 64 bits length was generated by using random number generator based on Rule 30 of cellular automata. 10 different nonce values were generated by using different seed values. Running times taken for ten sample executions are depicted in Fig. 4 and details are given as File S4.

The software performance of CA based nonce generation was implemented in 64-bit architecture. Algorithm was implemented in C and executed on an Intel i3 processor @2.00 GHz. Average time taken for nonce generation is 0.00052 s.

The initial key of 256 bits size and the generated nonce are fed as input to the ChaCha20 stream cipher and this generates the keystream.

**Figure 5:**
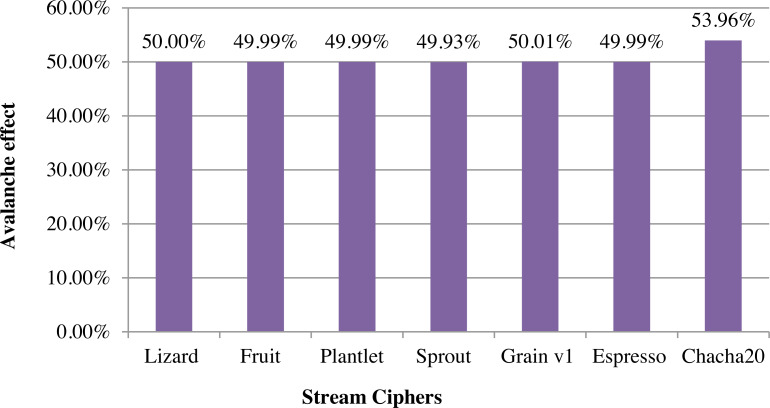
Comparison of stream ciphers based on key-keystream avalanche effect.

Comparison study was conducted by implementing ChaCha20 and other major lightweight stream ciphers in C. The avalanche effect based comparison between ChaCha20 stream cipher and other stream ciphers such as Lizard, Fruit, Plantlet, Sprout, Grain v1 and Espresso based on key and keystream is given in Fig. 5. Avalanche effect for stream ciphers is calculated based on the relation between key and keystream. The equation used for calculating avalanche effect is number of bits changed in keystream/number of bits in keystream. It checks the number of bits changed in the keystream generated when one bit in the initial key is changed. This ensures the diffusion of algorithm. A good encryption algorithm should have an avalanche effect greater than 50%. Here, ChaCha20 has an avalanche effect of 54%.

**Figure 6:**
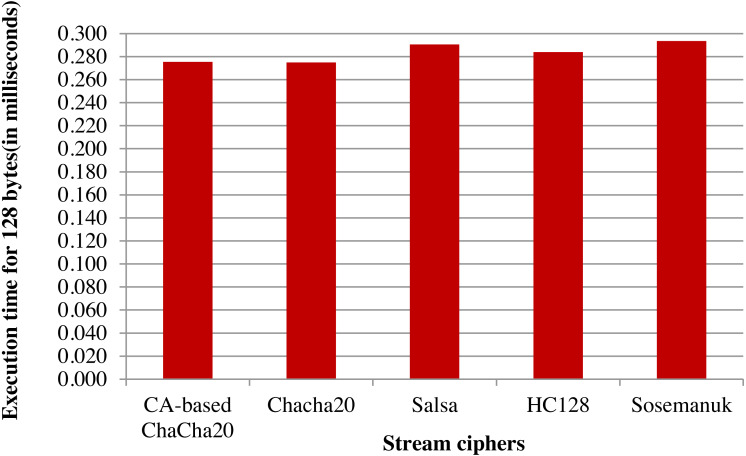
Comparison based on execution time taken for encryption.

**Figure 7:**
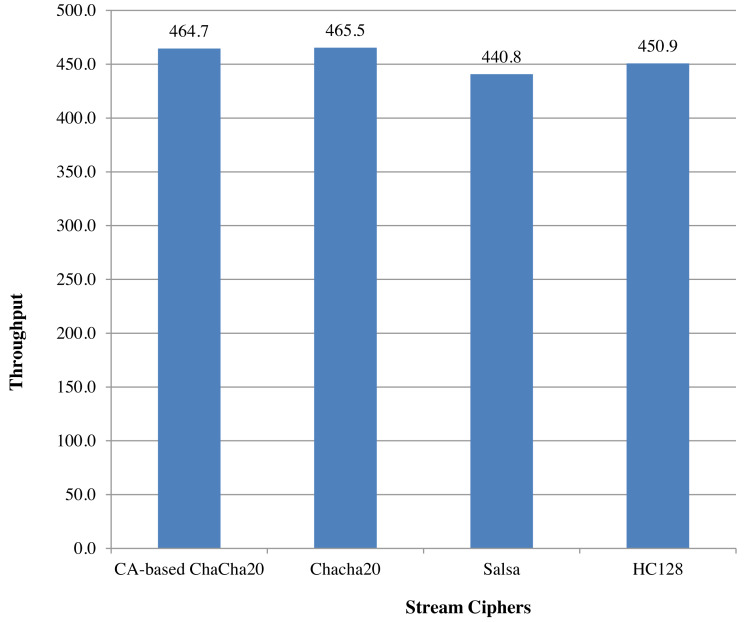
Comparison based on throughput PeerJ.

**Figure 8:**
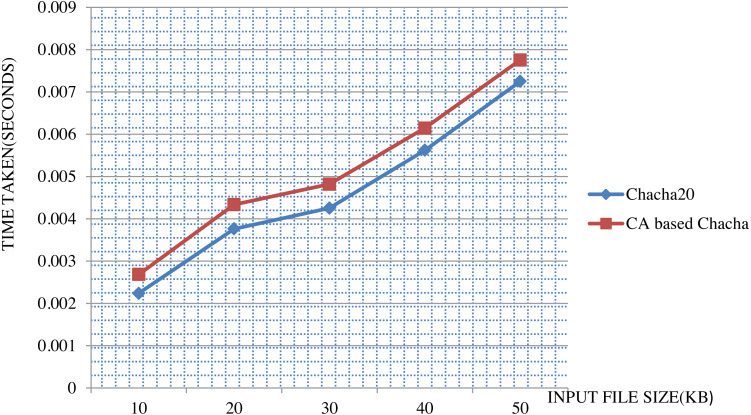
Comparison between ChaCha20 and CA-based ChaCha20.

An analysis of ChaCha20 cipher and CA-based ChaCha20 cipher, based on comparison with other lightweight stream ciphers in terms of execution time is given in Fig. 6. For the comparison, time taken in milliseconds for encrypting data of size 128 bytes was taken for each algorithm. For the same executions, throughput is calculated as number of Kilobytes encrypted per second. As indicated in the Fig. 7, ChaCha20 stream cipher has the best throughput compared to other stream ciphers. Throughput of ChaCha20 cipher is 465.4 and that of CA-based ChaCha20 cipher is 464.7. Though there is a slight difference between throughputs of CA-based ChaCha20 and ChaCha20, both these have proved to be far better in performance compared to the Salsa20 cipher with a throughput of 440.8. The CA-based ChaCha20 in which nonce was generated by using CA rule provides more security since the randomness of CA based number generation has been proved already. The randomness in selection of nonce value which is also known as initialization vector helps the data owner to improve data security. Also, this enhancement does not consume much CPU time for execution. Time taken for execution for different input file sizes is given in the Fig. 8.

**Figure 9:**
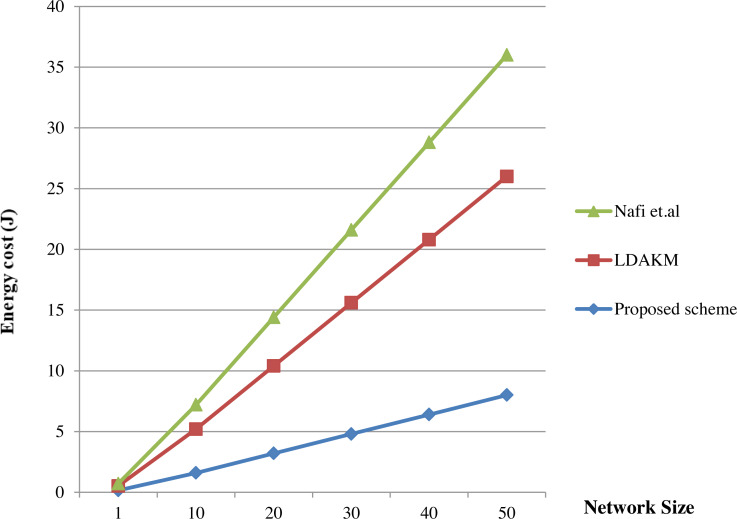
Comparison of key exchange methods.

In addition to the computational time, overhead of the proposed method was analyzed in terms of cost also. The authentication and key exchange phases were compared with the matrix based scheme in Nafi, Bouzefrane & Omar (2020) and Lightweight Device Authentication and Key Management Scheme (LDAKM) (Wazid et al., 2019). Computational cost of a security scheme is affected by the encryption steps, decryption steps and the hash computation steps used for authentication (Meulenaer et al., 2008; Wander et al., 2005). The energy cost of a hash operation is 40 mJ (Meulenaer et al., 2008). The key establishment and authentication phases of the proposed scheme cost less when compared to the similar phases in Nafi, Bouzefrane & Omar (2020) and Wazid et al. (2019). Authentication phase has four hash computations and hence it costs 160 mJ. The key exchange phase does not involve any hash computations. The protocols given in Nafi, Bouzefrane & Omar (2020) and Wazid et al. (2019) were already proved that they have lower cost and better performance compared to other related schemes. Now, it is proved that the proposed method is having lower cost compared to these two schemes and the same is depicted in Fig. 9.

## Security Analysis

Security analysis of the same was conducted using the Automated Validation of Internet Security Protocols and Applications (AVISPA) tool (Glouche et al., 2006). It is a security protocol verification and analysis tool in which new security protocol has to be written in High Level Protocol Specification Language (HLPSL) and then fed as input to the tool. Security of the given protocol will be analyzed with the built-in backend compilers. AVISPA consists of four different back-end compilers: On-the-fly Model-Checker (OFMC), Constraint-Logic-based Attack Searcher (CL-AtSe) , SAT-based Model-Checker(SATMC) and Tree Automata based on Automatic Approximations for the Analysis of Security Protocols (TA4SP). Different phases of the proposed model have been written in HLPSL and tested in AVISPA. The HLPSL representation snippet of the mutual authentication phase is provided as File S6. These HLPSL codes were compiled with the backend compilers provided in AVISPA. The proposed protocol was proved to be “SAFE” under OFMC, “SAFE” under ATSE, INCONCLUSIVE under SATMC and INCONCLUSIVE under TA4SE compilers. OFMC backend compiler verifies the protection status of protocol against passive intruder and here, this method was proved to be “SAFE”. Results from OFMC and ATSE compilers prove that, proposed method is safe from possible passive and active attacks like man in the middle attack, replay etc.

The proposed method is safe against almost all kinds of attacks with multiple levels of encryption and with the default security mechanisms in blockchain.

• Replay attack: Message exchanges in mutual authentication phase are based on timestamp values. This will provide protection from replay attacks. On receiving the message, the gateway node will calculate the difference between received timestamp and embedded sent timestamp value. This difference should not exceed an agreed value.
• Brute Force attack: The avalanche effect of the lightweight cipher used in the proposed method was calculated and good results were obtained. The purpose of Avalanche test itself is to prevent real-time attacks such as Brute Force attacks.
• Man in the middle attack: Even though a third party stands in between, it is not possible to involve in the communication because, the exchange of master key depends on private key matrices. Since the attacker is not aware of the private key values of involved parties, master key can be shared only between properly authenticated users. Blockchain members are authenticated by the peer participants for every communication.
• Eavesdropping attack: Network used is based on Dolev-Yao model (Dolev D. Yao, 1983). This means, all message transmissions over the network are visible to an untrusted adversary also. Even though there is an unintended observer, that entity will never be able to retrieve the two keys involved in data protection. The adversary is not aware of the previous value of K_GA_ and hence, it is not possible for him to get the new key value. Furthermore, all the transmissions between the device and the gateway are encrypted with the current session group key. In addition to this encryption, the CA based random number generation makes it infeasible to predict the keystream generated. Regarding the cloud data protection, data confidentiality is again achieved by using either asymmetric encryption or attribute based encryption based on the importance level of data.
• Password guessing attack: Since the authentication does not deal with passwords, the system is resistant against such attacks also. Guessing the secret key is applicable if same value is used for all session encryptions. Proposed method relies on dynamic values of key involved. Each session is initiated with a mutual authentication phase and hence, only authenticated entities will get the secret values. During the mutual authentication phase, guessing of nonce is also not possible because the attacker does not know the pre-shared hash function.
• Insider attack: The device which has to upload the data to cloud has to undergo a registration phase with the gateway node during which the device specific credentials will be passed. Also mutual authentication phase is completed between gateway node and the device each time a session is started. Hence, the proposed method is free from insider attacks.
• Known-key attack: The method is free from known key based attacks because, the encryption of data is done using dynamically generated session keys. Also, the key generation procedure of ChaCha20 cipher has proved to be unbreakable till date.

## Conclusions

A fast and efficient architecture for providing security in IoT based networks was proposed. The system makes use of an improved version of secure lightweight cipher ChaCha20. The security is further enhanced by providing multiple levels of encryption based on a dynamic session key. Mutual authentication ensures protection to each device involved in communication. Conventional IoT systems face some major security challenges which can be solved by changing to a distributed environment. The use of blockchain ensures the authentication and message integrity of data. Blockchains are suitable for developing a decentralized IoT network. As security goals are achieved in blockchain by default, integrating IoT with blockchain provides better security in terms of authentication, integrity of sensed data, etc. The proposed system was tested using analysis tool AVISPA and found to be safe. A comparison of CA-based ChaCha20 with other stream ciphers based on execution time, throughput and avalanche effect was also conducted and proved to be better in all these aspects. Moreover, the computational overhead of the proposed method was also analyzed. Further enhancements can be done related to cloud storage optimizations.

##  Supplemental Information

10.7717/peerj-cs.989/supp-1 Supplemental Information 1 Code for implementation of blockchain based healthcare application in Hyperledger Fabric Code for smart contracts in Hyperledger Fabric Platform for healthcare application.

10.7717/peerj-cs.989/supp-2 Supplemental Information 2 Screenshot of data access by doctor in Hyperledger Fabric

10.7717/peerj-cs.989/supp-3 Supplemental Information 3 Lightweight Ciphers based on NIST and eSTREAM project results

10.7717/peerj-cs.989/supp-4 Supplemental Information 4 10 executions of CA based random number generation

10.7717/peerj-cs.989/supp-5 Supplemental Information 5 Messages in mutual authentication phase

10.7717/peerj-cs.989/supp-6 Supplemental Information 6 A snippet of the HLPSL representation of proposed mutual authentication phase

## Additional Information and Declarations

### Data Availability

The following information was supplied regarding data availability: The data and codes are available in the Supplementary File.
