# Supplementary material for: An efficient multilevel security architecture for blockchain-based IoT networks using principles of cellular automata
Source: PeerJ Comput Sci. 2022 May 25;8:e989. doi: 10.7717/peerj-cs.989 (PMC9202632; doi:10.7717/peerj-cs.989)
Supplement: Supplemental Information 2 [file peerj-cs-08-989-s002.pdf]

```
Creating channel 'mychannel'...
Successfully created channel 'mychannel'
Joining Org1 peers to the channel...
Successfully joined peers to channel 'mychannel'
Joining Org2 peers to the channel...
Successfully joined peers to channel 'mychannel'
Installing chaincode on Org1 peer...
Successfully installed chaincode in peer 'localhost:7051'
Installing chaincode on Org2 peer...
Successfully installed chaincode in peer 'localhost:9051'
Instantiating chaincode...
Chaincode instantiation successfull
Enrolling patient 'Johny' on Org1...
Successfully enrolled user 'Johny' [80 97 116 105 101 110 116 32 69 110 114 111
108 108 101 100 32 83 117 99 99 101 115 115 102 117 108 108 121 46]
Enrolling doctor 'Alice' on Org2...
Successfully enrolled user 'Alice' [68 111 99 116 111 114 32 69 110 114 111 108
108 101 100 32 83 117 99 99 101 115 115 102 117 108 108 121 46]
Calling 'addDevice' chaincode function by Johny...
Device enrolled successfully

Calling 'queryData' chaincode function by Johny...
{"Devices":[{"DeviceID":"DEVICE123XYZ","MasterKey":"MASTERKEY","RequestedBy":nul
l,"SharedWith":[]}],"Id":"Johny","PrivateMatrix":"[[218, 188, 131, 32],[40, 42,
22, 5],[27, 28, 17, 4],[6, 5, 4, 1]]","Type":"Patient"}

Calling 'requestData' chaincode function by Alice...
Successfully sent request.

Calling 'getAllDoctors' chaincode function by Alice...
[{"Key":"Alice", "Record":{"Department":"Cardiology","Id":"Alice","PrivateMatrix
":"abcd678fghj","SharedData":[],"Type":"Doctor"}}]

Calling 'getAllPatient' chaincode function by Alice...
[{"Key":"Johny", "Record":{"Devices":[{"DeviceID":"DEVICE123XYZ","MasterKey":"MA
STERKEY","RequestedBy":["Alice"],"SharedWith":[]}],"Id":"Johny","PrivateMatrix":
"[[218, 188, 131, 32],[40, 42, 22, 5],[27, 28, 17, 4],[6, 5, 4, 1]]","Type":"Pat
ient"}}]
```

Screenshot of data access by doctor in Hyperledger Fabric
