# Supplementary material for: An efficient multilevel security architecture for blockchain-based IoT networks using principles of cellular automata
Source: PeerJ Comput Sci. 2022 May 25;8:e989. doi: 10.7717/peerj-cs.989 (PMC9202632; doi:10.7717/peerj-cs.989)
Supplement: Supplemental Information 3 [file peerj-cs-08-989-s003.docx]

| **Stream Ciphers** | **Block Ciphers** |
| --- | --- |
| Grain v1,  F-FCSR-H v2,  MICKEY v2Rabbit ,  HC-128,  Salsa20/12,  Sosemanuk,  CryptMT, Elephant  Dragon | PRESENT,  CLEFIA,  ASCON,  GIFT,  Photon-Beetle,  SIMON,SPECK |
