# Supplementary material for: An efficient multilevel security architecture for blockchain-based IoT networks using principles of cellular automata
Source: PeerJ Comput Sci. 2022 May 25;8:e989. doi: 10.7717/peerj-cs.989 (PMC9202632; doi:10.7717/peerj-cs.989)
Supplement: Supplemental Information 4 [file peerj-cs-08-989-s004.docx]

| Sl.No. | Initial seed | Nonce generated | Time taken  (Seconds) |
| --- | --- | --- | --- |
| 1 | 1234523 | 2589950946407450 | 0.000447 |
| 2 | 1436526 | 2721804441115020 | 0.000572 |
| 3 | 55246 | 5593811780602660 | 0.000564 |
| 4 | 41247 | 607195510575042 | 0.000525 |
| 5 | 2783526 | 3939014422912540 | 0.000505 |
| 6 | 4185526 | 7587125081972140 | 0.000578 |
| 7 | 3245526 | 1296497774919630 | 0.00054 |
| 8 | 552612346 | 5317958170444910 | 0.000454 |
| 9 | 3226526 | 5695001779130750 | 0.000526 |
| 10 | 1783526 | 5547601352180820 | 0.000491 |
