# Supplementary material for: An efficient multilevel security architecture for blockchain-based IoT networks using principles of cellular automata
Source: PeerJ Comput Sci. 2022 May 25;8:e989. doi: 10.7717/peerj-cs.989 (PMC9202632; doi:10.7717/peerj-cs.989)
Supplement: Supplemental Information 5 [file peerj-cs-08-989-s005.docx]

*Message_1 = E({IDa, IDg, M1, M2, TSi}, K_GA_^i^)*

*Where, E is the encryption algorithm*

*K_GA_^i^ is the i^th^ group key*

*IDa is the unique identity of device, A.*

*IDg is the identity of gateway node, G.*

*M1 = H(IDa || TSi) ⊕ Na*

*M2* ***=*** *H(IDa || IDg || TSi, Na)*

*Message_2 = E({IDg,IDa,M3,M4,Ng,TSj}, K_GA_)*

*Where, IDg, IDa, Na, H, ⊕ and || are same as that of Message_1 components.*

*M3 = H(IDg || TSj) ⊕ Ng*

*M4 = H(IDg || IDa || TSj || Ng ||Na)*

*H is the hash function commonly agreed between A and G.*

*TSi is the Timestamp value when A sends the Message_1. Na is the nonce value generated randomly by the device, A. ‘⊕’ denotes the XOR operator and ‘||’ denotes concatenation. TSj is the timestamp at which G sends Message_2 to A. Ng is the nonce generated randomly by the gateway node, G.*
