# Supplementary material for: An efficient multilevel security architecture for blockchain-based IoT networks using principles of cellular automata
Source: PeerJ Comput Sci. 2022 May 25;8:e989. doi: 10.7717/peerj-cs.989 (PMC9202632; doi:10.7717/peerj-cs.989)
Supplement: Supplemental Information 6 [file peerj-cs-08-989-s006.pdf]

```
role gateway(A,G:   agent,
Kga:   symmetric_key,  H:        hash_func,
SND,RCV:  channel(dy))
played_by G def=
%% Variables declaration
local
State: nat,
IDa,IDg,TS1, TS2, Km: text,
M1, M2, M3,M4 : text,
Rn,Na,Ng    :message
const m:     protocol_id
init State := 1
%%Transition rulesof steps taken by gateway node in HLPSL
transition
1. State = 1/\RCV({IDa'.IDg'.M1'.M2'.TS1'}_Kga)        /\M2'=H(IDa'.IDg'.TS1'.xor(H(IDa'.TS1'),M1') ) =|>
State' := 2 /\Na' := xor(H(IDa'.TS1'),M1') /\Ng' :=new()/\M3' := xor(H(IDg.TS2),Ng') /\M4' :=
H(IDg.IDa'.TS2.Ng'.Na')/\SND({IDg.IDa'.M3'.M4'.Ng'.TS2}_Kga)
    2.     State = 3  /\RCV({IDa'.IDg'.Rn'.Km'}_Kga)
```
